# Combined unicompartmental knee arthroplasty and ACL reconstruction for medial knee osteoarthritis complicated by ACL insufficiency: superior early functional recovery with LARS artificial ligament and comparable mid-term results versus autologous tendon

**DOI:** 10.3389/fmed.2026.1935913

**Published:** 2026-08-19

**Authors:** Ke He, Ling Yuan, Zhenyu Luo, Yuan Li, Xu Peng, Guanjun Sun, Yi Yin, Yu Leng, Xiaoyi Jiao

**Affiliations:** 1 Department of Joint and Sports Medicine, Suining Central Hospital, Suining, Sichuan, China; 2 Department of Health Management, Suining Central Hospital, Suining, Sichuan, China

**Keywords:** ACL deficiency, anterior cruciate ligament reconstruction, autologous tendon graft, LARS artificial ligament, unicompartmental knee arthroplasty

## Abstract

**Background:**

To evaluate the outcomes of fixed-bearing medial unicompartmental knee arthro plasty (UKA) combined with anterior cruciate ligament (ACL) reconstruction using either a LARS artificial ligament or autologous tendon graft, compared with isolated UKA in ACL-intact knees.

**Methods:**

Patients who underwent UKA combined with ACL reconstruction from January 2019 to December 2021 were retrospectively reviewed. They were divided into the LARS group (L-UKA, *n* = 20) and the autologous tendon group (A-UKA, *n* = 20). A contemporaneous cohort of patients who underwent isolated UKA with an intact ACL was matched at a 1:1:1 ratio (UKA, *n* = 20). Range of motion (ROM), Knee Society Score (KSS), Oxford Knee Score (OKS), and Forgotten Joint Score (FJS) were compared. Radiographic parameters included the hip-knee-ankle angle (HKA), posterior tibial slope (PTS), and anterior tibial translation (ATT).

**Results:**

The mean follow-up was 61.07 ± 3.90 months. Baseline characteristics were comparable among the three groups. At final follow-up, ROM, KSS and OKS improved significantly in all groups compared with preoperative values (*P* < 0.01), with no significant intergroup differences (*P* > 0.05). Final HKA, PTS, and ATT were also comparable among groups. Preoperative ATT was significantly greater in the L-UKA and A-UKA groups than in the isolated UKA group (*P* < 0.01), but normalized postoperatively. At 1 month, ROM and KSS were significantly better in the L-UKA group than in the A-UKA group (*P* < 0.05). Two patients in the A-UKA group had donor-site pain. No implant loosening or lateral compartment OA progression occurred, and implant survivorship was 100%.

**Conclusion:**

UKA combined with ACL reconstruction using either LARS artificial ligament or autologous tendon graft effectively corrected ATT and achieved mid-term outcomes comparable to isolated UKA. LARS avoided donor-site morbidity and provided better early functional recovery, although its long-term safety requires further validation.

## 1 Introduction

Unicompartmental knee arthroplasty (UKA) is an established surgical option for end-stage medial compartment osteoarthritis (OA), offering advantages such as bone preservation, faster recovery, and more physiological knee kinematics compared with total knee arthroplasty (TKA) (1–3). Early work by Kozinn and Scott emphasized that successful UKA depends on strict patient selection, in which an intact anterior cruciate ligament (ACL) is considered a key requirement. ACL deficiency may lead to anterior knee instability, altered tibiofemoral kinematics, and increased risk of polyethylene wear or implant loosening (4). Therefore, ACL insufficiency has traditionally been regarded as a relative or absolute contraindication for UKA in many clinical guidelines (5, 6).

In recent years, this traditional indication has been increasingly challenged. Studies by Du et al. and Guo et al. have reported that UKA can achieve acceptable mid- to long-term outcomes in carefully selected patients with ACL deficiency and isolated medial compartment OA (7, 8). Notably, these patients were typically older, had low functional demands, and presented with pain as the predominant symptom rather than instability. For relatively younger, more active patients presenting with ACL rupture in the setting of OA, who experience both pain and instability as their predominant symptoms and have high functional demands, however, persistent anterior tibial translation and abnormal joint loading patterns remain concerns, which may affect knee biomechanics and potentially contribute to progression of lateral compartment degeneration (9).

To address these issues, some authors have proposed combining UKA with anterior cruciate ligament reconstruction (ACLR) as a joint-preserving strategy aimed at restoring both compartmental pathology and global knee stability (10). A recent systematic review by Vosoughi et al. has suggested that this combined procedure may provide satisfactory functional outcomes in selected patients (11). As a result, UKA combined with ACLR has been increasingly considered a potential option for younger, more active patients with ACL-deficient medial compartment OA. However, there is still no consensus regarding whether anterior cruciate ligament reconstruction is necessary in this setting (12, 13).

Regarding graft selection, autologous hamstring tendon grafts remain widely used in ACL reconstruction due to their reliable biological incorporation and long-term remodeling potential (14). However, donor-site morbidity and the graft ligamentization process may delay early postoperative recovery. In contrast, synthetic ligaments such as the Ligament Advanced Reinforcement System (LARS) provide immediate mechanical stability and allow early rehabilitation, although concerns remain regarding long-term biocompatibility and potential synovial complications (15).

Although extensive literature has compared LARS versus autografts in isolated ACLR, these conclusions cannot be directly extrapolated to the unique setting of UKA combined with ACLR. Graft selection in this combined procedure faces some distinct challenges: First of all, the tibial tunnel must “share space” with the UKA keel, necessitating a more lateral and vertical trajectory; LARS, with its standardized diameter and immediate mechanical strength, offers greater predictability in this constrained space than autografts that rely on ligamentization and exhibit insufficient early strength. Secondly, Fixed-bearing prostheses alter the tibiofemoral center of rotation and contact points, compelling the reconstructed ACL to bear the primary restraining load for controlling anterior tibial translation and rotational instability–a mechanical demand fundamentally distinct from isolated ACLR. Consequently, the deterministic mechanical properties of LARS may be more suitable for this non-physiological kinematic environment. Then, with the posterior tibial slope (PTS) strictly controlled within a 4°–5° safety window, graft tension is highly sensitive to minor angular variations; autografts exhibit poor tolerance to PTS-related loads during the early strength nadir of ligamentization, whereas the zero-creep property of LARS not only eliminates the confounding effect of graft laxity on anterior tibial translation (ATT) outcomes but also better accommodates early rehabilitation under precise PTS control. Therefore, this study aims to investigate whether LARS offers superior clinical outcomes compared to autografts under these prosthetic constraints, addressing a specific research gap in graft selection for combined procedures.

## 2 Materials and methods

### 2.1 Study design and group allocation

This single-center retrospective matched cohort study included patients who underwent fixed-bearing medial unicompartmental knee arthroplasty (UKA) combined with anterior cruciate ligament reconstruction (ACLR) between January 2019 and December 2021 at the Department of Joint and Sports Medicine, Suining Central Hospital.

Patients were assigned to three groups according to anterior cruciate ligament status and surgical strategy: the L-UKA group (UKA combined with ACLR using a Ligament Advanced Reinforcement System [LARS] artificial ligament), the A-UKA group (UKA combined with ACLR using autologous hamstring tendon grafts), and the UKA group (isolated UKA with intact ACL, serving as the control group). To ensure comparability among groups and reduce potential selection bias, a 1:1:1 matched cohort design was applied. Matching variables were defined *a priori* and included age (±3 years), sex, body mass index (BMI), surgical side, Kellgren–Lawrence grade, and major comorbidities (including hypertension and diabetes). After matching, baseline characteristics were well balanced across the three groups. A total of 60 patients were included in the final analysis, with 20 patients in each group.

The study was approved by the Ethics Committee of Suining Central Hospital (Approval No. XJS2020-064-01) and conducted in accordance with the Declaration of Helsinki. Written informed consent was obtained from all participants.

### 2.2 Inclusion and exclusion criteria

Patients in the L-UKA and A-UKA groups met the following inclusion criteria: (1) diagnosis of anterior cruciate ligament (ACL) deficiency confirmed by clinical examination, including a Lachman test grade ≥2+ and a positive pivot-shift test, as well as magnetic resonance imaging (MRI) findings; (2) age ≤65 years; (3) isolated medial compartment osteoarthritis classified as Kellgren–Lawrence grade III or IV; (4) preserved lateral and patellofemoral compartments without advanced degenerative changes; (5) preoperative flexion contracture ≤10° and varus deformity ≤15°, both correctable under stress radiography; and (6) preoperative range of motion (ROM) ≥ 90°. Patients in the UKA group met the same radiographic and functional criteria, except for having an intact ACL confirmed by clinical examination and MRI.

**FIGURE 1:**
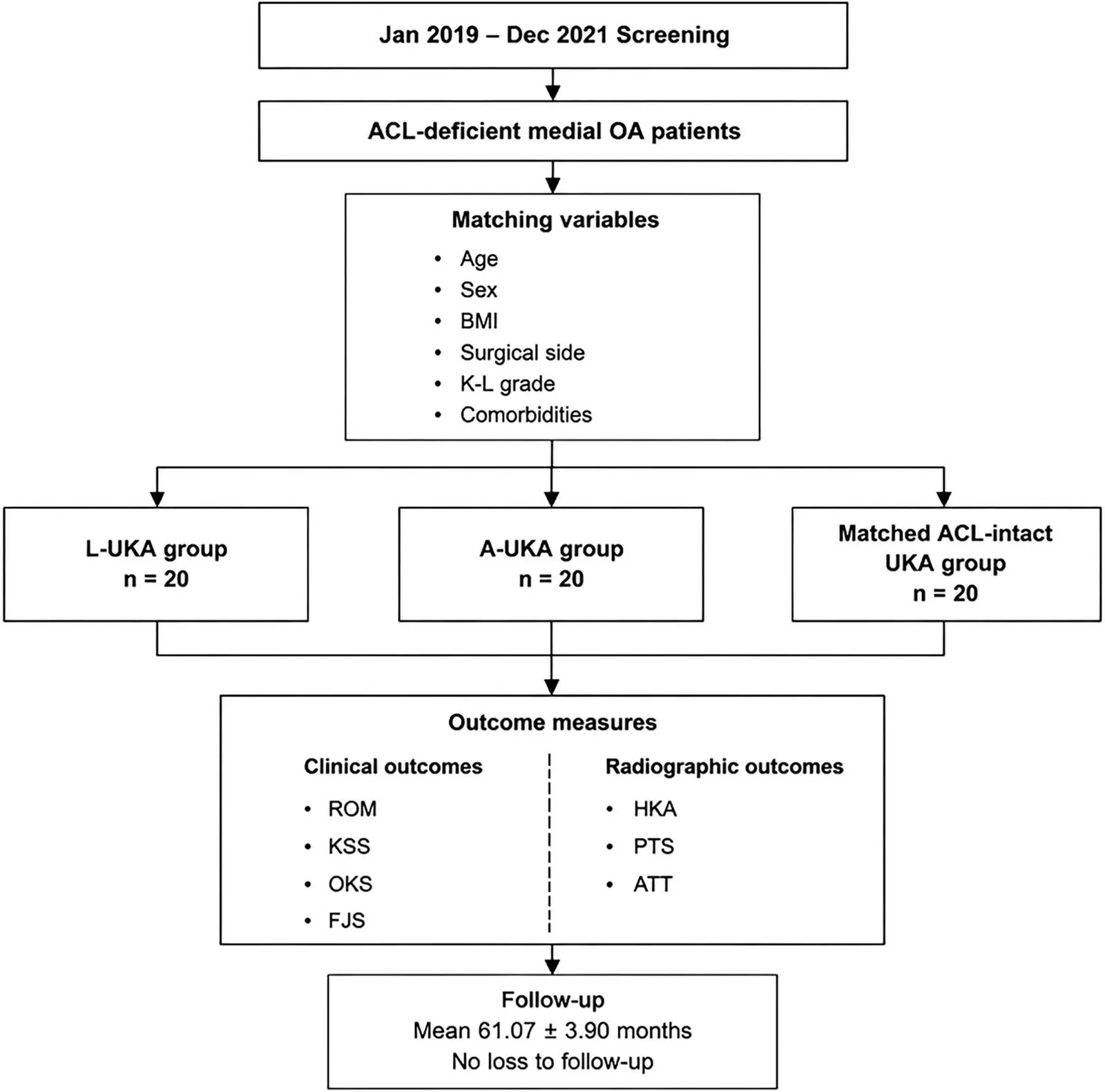
Flowchart of participant screening and grouping in this study. ROM, range of motion; KSS, Knee Society Score; OKS, Oxford Knee Score; HKA, hip-knee-ankle angle; PTS, posterior tibial slope; ATT, anterior tibial translation; FJS, Forgotten Joint Score.

The exclusion criteria for all groups were as follows: inflammatory arthritis; concomitant posterior cruciate ligament or multiligament injuries; body mass index (BMI) > 35 kg/m^2^; history of septic arthritis, severe osteoporosis, or neuromuscular disorders affecting knee function; and incomplete clinical or imaging follow-up data (Figure 1).

### 2.3 Surgical procedures

All procedures were performed by the same team of senior joint surgeons, using general anesthesia combined with a femoral nerve block. The patients were positioned supine with a pneumatic tourniquet applied to the proximal thigh. Following anesthesia induction, the integrity of the ACL was re-evaluated via the Lachman test and the pivot-shift test. The medial compartment was exposed using a standard anteromedial knee approach, followed by resection of the degenerated medial meniscus and osteophytes. Femoral and tibial osteotomies were performed according to the fixed-bearing medial UKA protocol, controlling the posterior tibial slope (PTS) at 4–5°. Tibial vertical and horizontal cuts, along with keel slot preparation, were made while preserving the anatomical footprint of the ACL tibial attachment. Following trial reduction, gap balance and ligament tension were assessed throughout knee flexion and extension to confirm component fit and joint stability. In the isolated UKA group, only the arthroplasty steps were completed with careful preservation of the native ACL structure.

**FIGURE 2:**
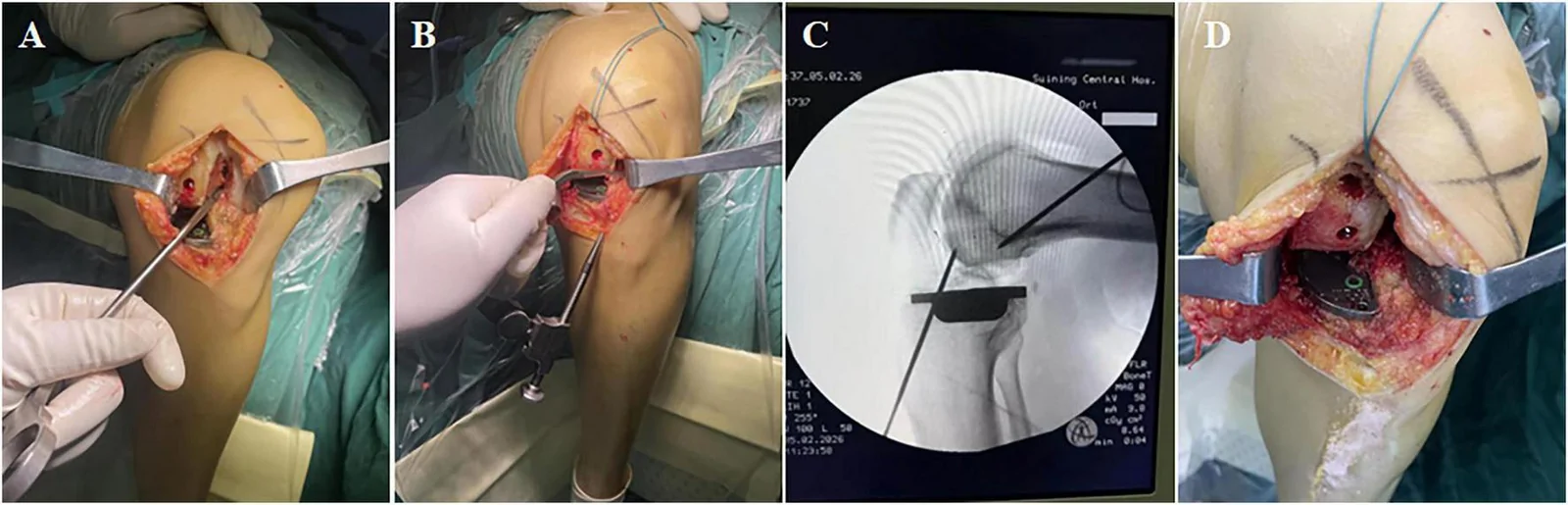
Intraoperative procedures for ACL tunnel creation. **(A)** Femoral tunnel preparation; **(B)** Tibial tunnel preparation; **(C)** Intraoperative C-arm fluoroscopy for tunnel positioning; **(D)** Complete tunnel configuration after drilling.

For the A-UKA group, the incision was extended distally to harvest the ipsilateral semitendinosus and gracilis tendons, which were prepared into a 4-strand or greater autograft. If the graft diameter was <8 mm, the proximal one-third to one-half of the ipsilateral peroneus longus tendon was added for reinforcement. In both the L-UKA and A-UKA groups, ACLR was performed using a quasi-isometric technique. The femoral tunnel was prepared with the knee flexed to approximately 120°. The center of the femoral tunnel was positioned slightly anterior to the level of the termination point of the lateral cartilage margin, with a 2 mm posterior wall retained. The center of the intra-articular opening of the tibial tunnel is located on the anterior slope of the intercondylar eminence, 2–3 mm medial to the anterior root of the lateral meniscus. Medial and lateral offset creates a transverse safety interval between the tunnel and the tibial keel groove. We routinely use a hollow tibial guide set at 55°–60° relative to the horizontal plane of the tibial plateau to create the tunnel path. Intraoperative real-time C-arm fluoroscopy is applied to adjust the trajectory of the tibial guide wire, ensuring a cancellous bone bridge of at least 4 mm between the medial wall of the tunnel and the lateral edge of the prosthesis keel to prevent tunnel-keel coalescence and impingement (Figure 2). After preparing the tunnels, the LARS artificial ligament or autograft was introduced into them. Trial components and polyethylene liners were then inserted to verify the absence of impingement and dislocation risk and to confirm the quasi-isometric behavior of the graft during knee motion. The final prostheses were then implanted. Graft fixation was performed at 30° of knee flexion with the tibia in neutral alignment. The L-UKA group used titanium interference screws for fixation, while the A-UKA group used a suspensory titanium plate for fixation of the femur and a bioabsorbable interference screw for fixation of the tibia.

### 2.4 Postoperative rehabilitation protocol

All three groups followed a standardized postoperative rehabilitation protocol. Antibiotics were administered routinely for 24 h after surgery; rivaroxaban was initiated 6 h after surgery to prevent deep vein thrombosis (continued until 21 days postoperatively in patients without contraindications); and non-steroidal anti-inflammatory drugs (NSAIDs) were administered routinely for multimodal pain management. On the first postoperative day, drainage tubes were removed, and follow-up X-rays of the lower extremities and venous color Doppler ultrasounds were performed. Following approval by the rehabilitation physician, patients in the L-UKA and UKA groups began full weight-bearing ambulation with a walker and unrestricted full range of motion (ROM) exercises for the knee on the first postoperative day. Patients in the A-UKA group were also encouraged to engage in early mobilization. However, for activities involving extreme flexion or high-load movements, the rehabilitation therapist provided individualized protective guidance based on muscle control.

### 2.5 Outcome measures

**FIGURE 3:**
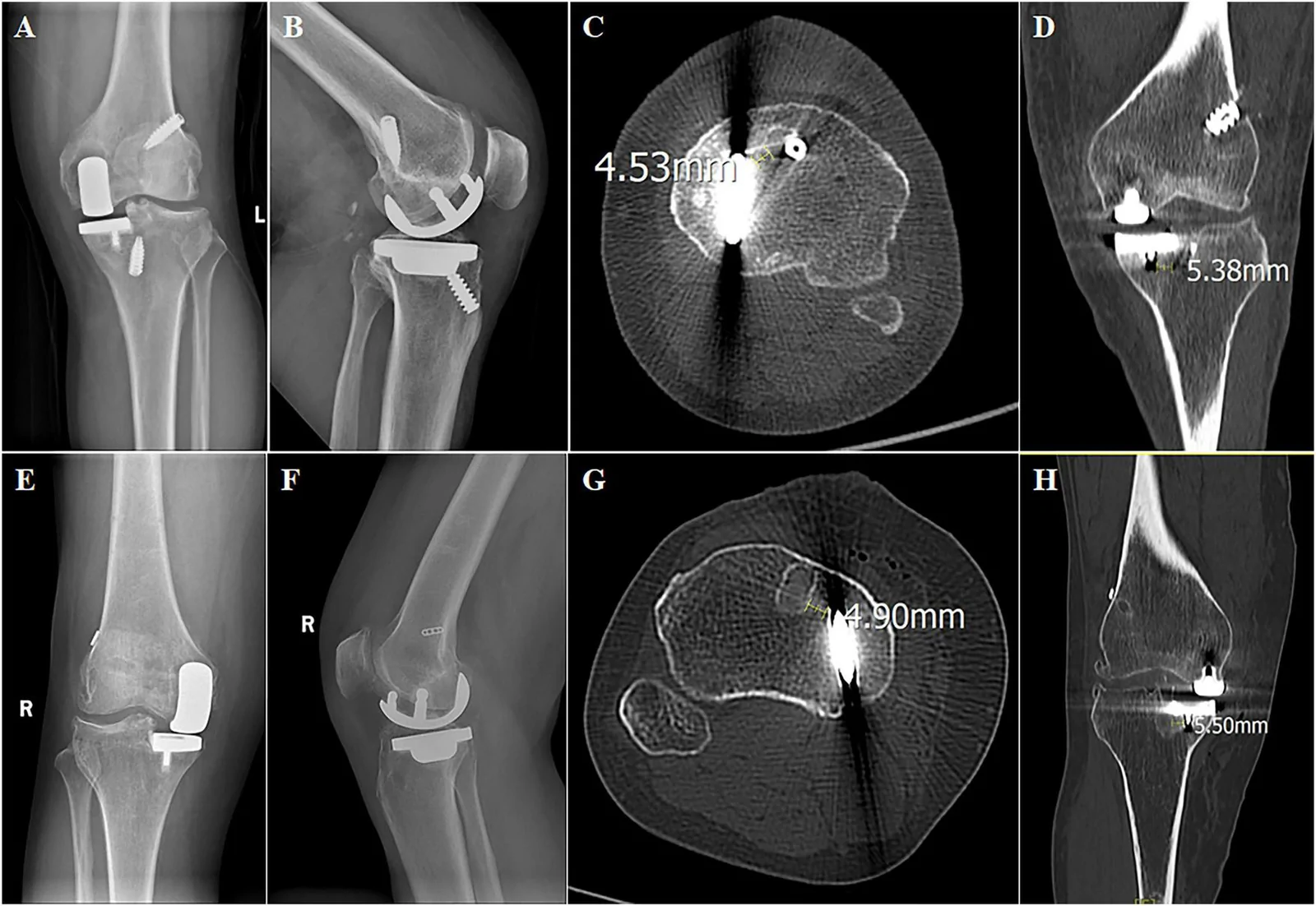
Postoperative weight-bearing radiographs and CT images at final follow-up. **(A,B)** Anteroposterior and lateral radiographs of the left knee in the L-UKA group; **(C,D)** axial and coronal CT images of the same knee in the L-UKA group, showing the measured bone bridge values between the prosthetic keel and the bone tunnel (4.53 mm and 5.38 mm, respectively), with no evidence of bone cement leakage; **(E,F)** anteroposterior and lateral radiographs of the right knee in the A-UKA group; **(G,H)** axial and coronal CT images of the same knee in the A-UKA group, showing the measured bone bridge values between the prosthetic keel and the bone tunnel (4.90 mm and 5.50 mm, respectively), with no evidence of bone cement leakage.

**FIGURE 4:**
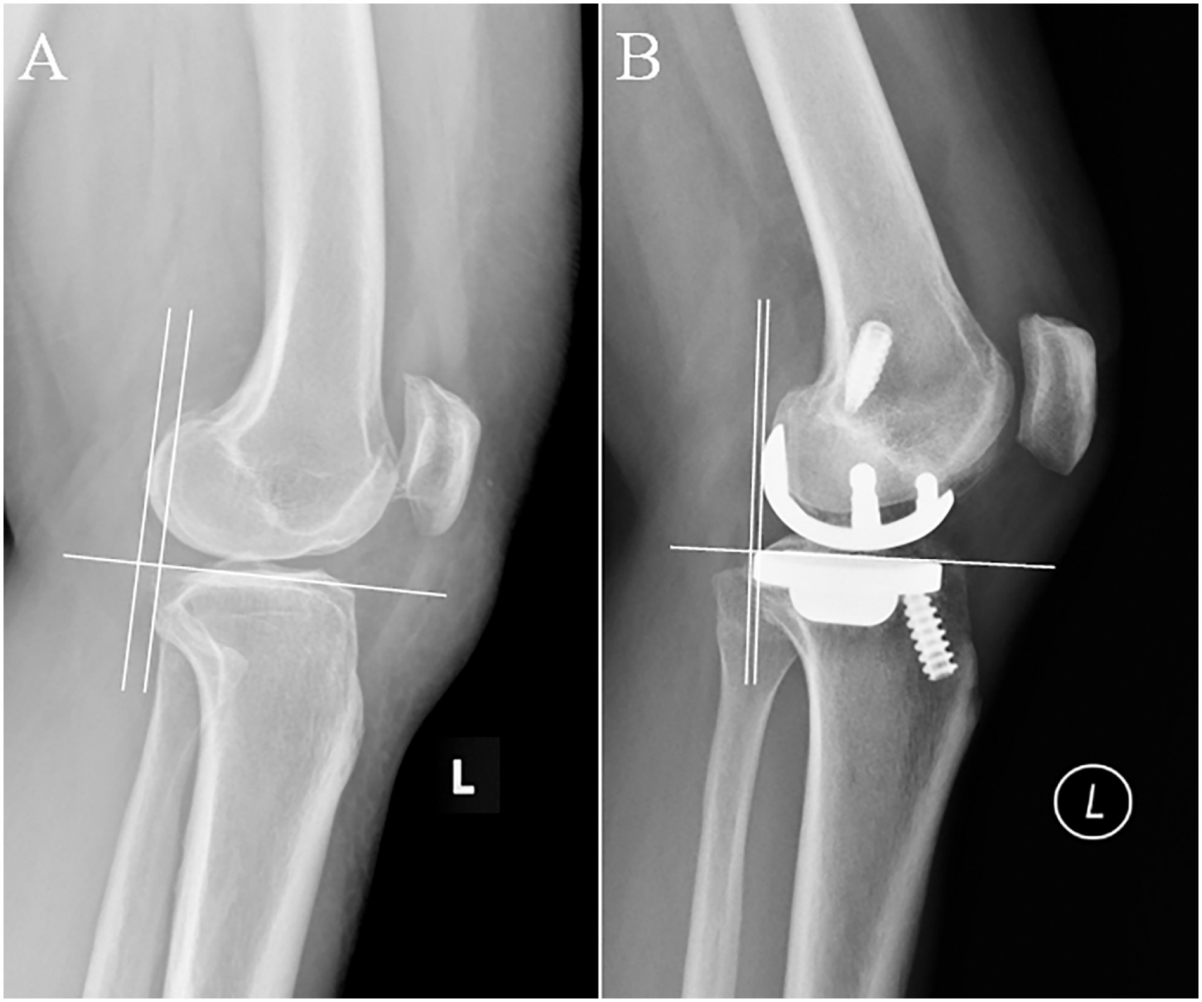
Schematic lateral radiographs illustrating the ATT measurement. **(A)** Preoperative lateral radiograph of the knee showing the reference lines for ATT measurement; **(B)** postoperative lateral radiograph of the knee showing the reference lines for ATT measurement.

Follow-up was conducted at 1, 3, 6, and 12 months postoperatively, and annually thereafter. The range of motion (ROM), the Knee Society Score (KSS), the Oxford Knee Score (OKS), and the Forgotten Joint Score (FJS) were recorded and compared between the preoperative assessment and the follow-ups. All ROM values represent passive range of motion, measured in supine position by the same team of standardized-trained assessors using a digital goniometer. The OKS used is the 12-item version, with each item scored on a scale of 0–4 (0 = no symptoms/best function; 4 = severe symptoms/worst function). The total score ranges from 0 to 48, with a lower score indicating better knee function. Complications such as poor wound healing, infection, graft failure, prosthesis loosening, and lateral compartment degeneration were closely monitored. Standardized weight-bearing radiographs and knee CT scans were obtained for all patients (Figure 3), and all images were assessed using the Picture Archiving and Communication System (PACS). Radiographic parameters included the hip-knee-ankle angle (HKA), posterior tibial slope (PTS), and anterior tibial translation (ATT). HKA was measured on full-length standing anteroposterior radiographs, and PTS was measured on standardized lateral radiographs. ATT was measured on standardized weight-bearing lateral radiographs using a method modified from previously described radiographic techniques for assessing anterior tibial translation (16). All X-rays were taken with the patient bearing weight on one leg and the knee flexed to 30°; measurements were taken using the posterior margin of the medial femoral condyle as a reference point, and the horizontal distance between the posterior cortex of the tibial plateau and the posterior contour of the femoral condyle was recorded (Figure 4). Each radiographic parameter was measured three times, and the average value was used for analysis. All measurements were performed independently by two orthopedic attending physicians who were not involved in the surgeries and were blinded to the clinical outcomes. The reliability of the measurements was also assessed.

### 2.6 Statistical methods

All clinical and radiological data were analysed using SPSS 26.0 (IBM Corp., Armonk, NY, USA). Continuous variables were first tested for normality using the Shapiro–Wilk test. Data that were normally distributed are presented as mean ± standard deviation, while data that were not normally distributed are presented as median and interquartile range. Categorical variables are presented as frequency and percentage. Within-group comparisons between the preoperative and postoperative periods were performed using the paired *t*-test for normally distributed data and the Wilcoxon signed-rank test for non-normally distributed data. Between-group comparisons of normally distributed continuous variables with homogeneous variances were analysed using one-way ANOVA, with *post hoc* comparisons performed using the Bonferroni method. Non-normally distributed or heteroscedastic variables were analysed using the Kruskal–Wallis H test, followed by Dunn’s *post hoc* test. The Pearson chi-square test or Fisher’s exact test was used for categorical variables. The inter-rater reliability of the radiographic measurements was assessed using the intraclass correlation coefficient (ICC). An ICC greater than 0.8 was considered to indicate good reliability. All tests were two-sided, and a *P*-value of less than 0.05 was considered statistically significant.

## 3 Results

All patients completed the scheduled follow-up, and no patients were lost to follow-up. The mean follow-up duration was 61.07 ± 3.90 months. After matching for baseline characteristics, including gender, age, BMI, side of surgery, K-L classification, and comorbidities (such as hypertension and diabetes), the three groups were well balanced and comparable (all *P* > 0.05) (Table 1). The ICC values ranged from 0.841 to 0.928, indicating satisfactory inter-observer agreement (Supplementary Table 1).

**TABLE 1:** Baseline characteristics.

Indicator	UKA group	L-UKA group	A-UKA group	Statistical value (F/χ ^2^)	*P*-value
Sex (male/female, *n*)	9/11	13/7	10/10	1.60	>0.05
Age (years)	61.12 ± 3.31	59.97 ± 3.19	60.54 ± 2.96	0.65	>0.05
BMI (kg/m^2^)	25.13 ± 1.8	24.85 ± 2.1	25.66 ± 3.19	0.42	>0.05
Operative side (left/right)	13/7	8/12	9/11	3.15	>0.05
Kellgren-Lawrence grade (grade III/IV)	10/10	12/8	9/11	0.82	>0.05
Follow-up (months)	59.94 ± 5.01	62.17 ± 2.36	61.11 ± 3.72	1.68	>0.05

### 3.1 Clinical functional assessment

**FIGURE 5:**
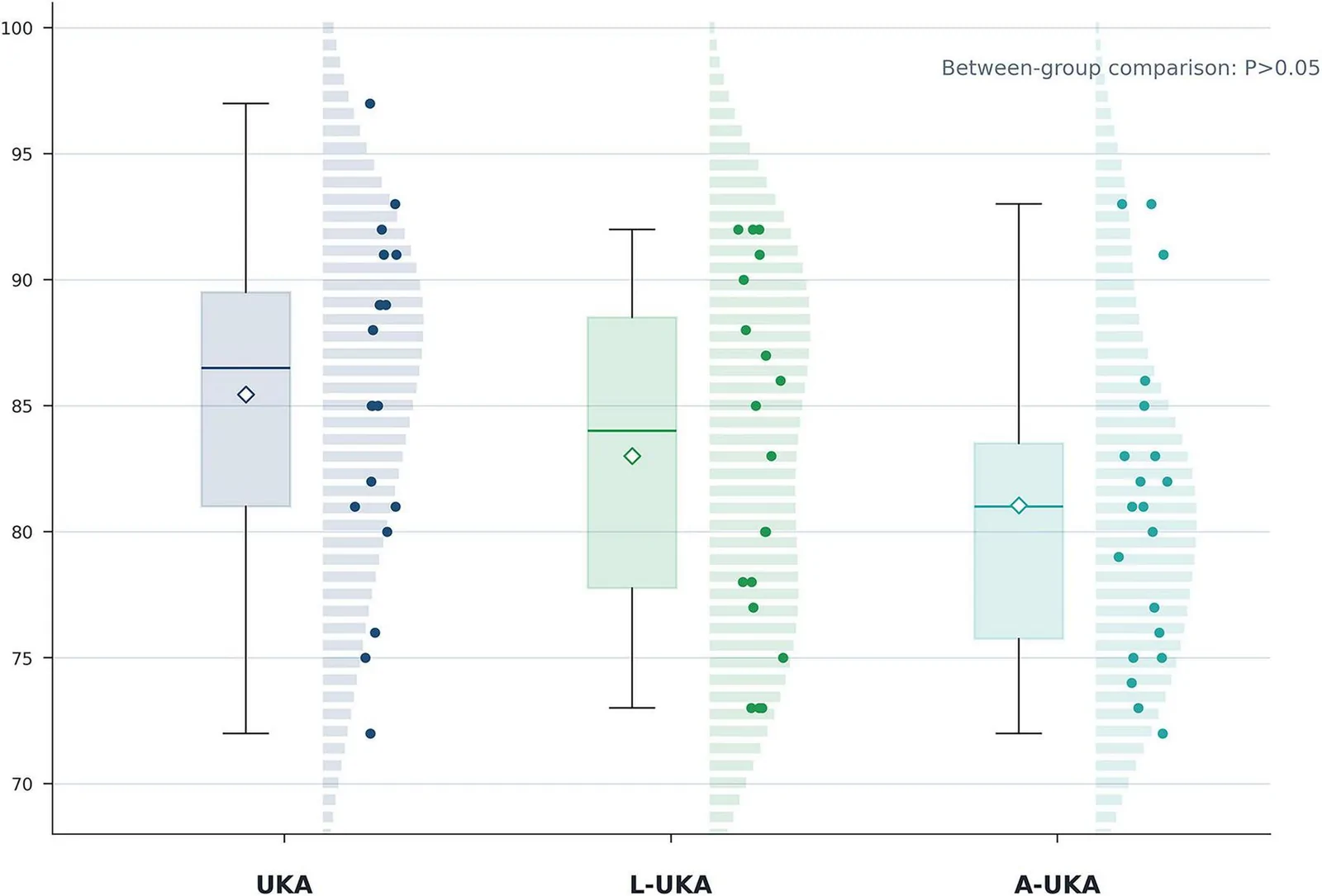
Distribution of FJS values across the three groups at final follow-up. No significant difference was detected in intergroup comparison (*P* > 0.05).

Preoperatively, there were no statistically significant differences in ROM, KSS scores, or OKS scores among the three groups of patients (*P* > 0.05) (Supplementary Table 2). At the final follow-up, all clinical outcome measures improved significantly in the three groups compared to preoperative levels (intra-group comparisons, *P* < 0.01) (Table 2). Intergroup comparisons at the final follow-up revealed no significant differences in ROM, KSS, or OKS scores (*P* > 0.05). Regarding FJS, both the L-UKA group (83.00 ± 6.86) and the A-UKA group (82.30 ± 6.12) achieved comparable levels to the isolated UKA group (86.70 ± 5.07), with no significant differences between groups (Table 3). These results suggest that both approaches can effectively maintain normal knee physiological movement patterns in UKA patients with ACL dysfunction and provide a sense of “joint amnesia” comparable to that achieved with isolated UKA (Figure 5).

**TABLE 2:** Intra-group comparisons of clinical functional indicators among the three patient groups at different follow-up time points.

Indicator	Procedure	T0 (M ± SD)	T1 (M ± SD)	T3 (M ± SD)	T0 VS. T1 (*P*-value)	T0 VS. T3 (*P*-value)	T1 VS. T3 (*P*-value)
ROM	UKA	103.30 ± 10.2	105.41 ± 2.00	126.13 ± 4.67	*P* > 0.05	*P* < 0.01	*P* < 0.01
	L-UKA	104.28 ± 6.62	103.30 ± 10.03	122.32 ± 6.50	*P* > 0.05	*P* < 0.01	*P* < 0.01
	A-UKA	106.63 ± 9.91	95.40 ± 9.72	125.52 ± 5.75	*P* < 0.05	*P* < 0.01	*P* < 0.01
KSS	UKA	65.95 ± 7.60	56.25 ± 5.99	83.70 ± 5.83	*P* < 0.01	*P* < 0.01	*P* < 0.01
	L-UKA	63.05 ± 8.58	54.85 ± 4.51	85.2 ± 6.19	*P* < 0.01	*P* < 0.01	*P* < 0.01
	A-UKA	67.20 ± 9.11	50.75 ± 4.77	81.60 ± 6.28	*P* < 0.01	*P* < 0.01	*P* < 0.01
OKS	UKA	35.10 ± 3.23	36.45 ± 3.44	20.15 ± 3.66	*P* > 0.05	*P* < 0.01	*P* < 0.01
	L-UKA	36.65 ± 3.63	37.10 ± 2.92	17.35 ± 4.42	*P* > 0.05	*P* < 0.01	*P* < 0.01
	A-UKA	34.95 ± 3.62	36.55 ± 3.46	19.25 ± 4.51	*P* > 0.05	*P* < 0.01	*P* < 0.01

ROM: range of motion; KSS: Knee Society Score; OKS: Oxford Knee Score. T0: preoperative; T1: 1 month postoperative; T2: 3 months postoperative; T3: final follow-up. *P* < 0.05 indicates a statistically significant difference.

**TABLE 3:** Intergroup comparisons of relevant indicators among the three groups at the time of the final follow-up.

Surgical procedure	ROM (M ± SD)	KSS (M ± SD)	OKS (M ± SD)	HKA (M ± SD)	PTS (M ± SD)	ATT (M ± SD)	FJS (M ± SD)
UKA	126.13 ± 4.67	83.70 ± 5.83	20.15 ± 3.66	177.32 ± 1.31	5.02 ± 0.91	0.48 ± 0.63	86.70 ± 5.07
L-UKA	122.32 ± 6.50	85.2 ± 6.19	17.35 ± 4.42	178.08 ± 1.20	4.53 ± 0.99	0.33 ± 0.66	83.00 ± 6.86
A-UKA	125.52 ± 5.75	81.60 ± 6.28	19.25 ± 4.51	177.80 ± 1.20	4.80 ± 1.03	0.42 ± 0.64	82.30 ± 6.12
*F*-value	2.59	1.76	2.30	1.93	1.25	0.28	3.04
*P*-value	*P* > 0.05	*P* > 0.05	*P* > 0.05	*P* > 0.05	*P* > 0.05	*P* > 0.05	*P* > 0.05

ROM: range of motion; KSS: Knee Society Score; OKS: Oxford Knee Score; HKA: hip-knee-ankle angle; PTS: posterior tibial slope; ATT: anterior tibial translation; FJS: Forgotten Joint Score. *P* < 0.05 indicates a statistically significant difference.

**FIGURE 6:**
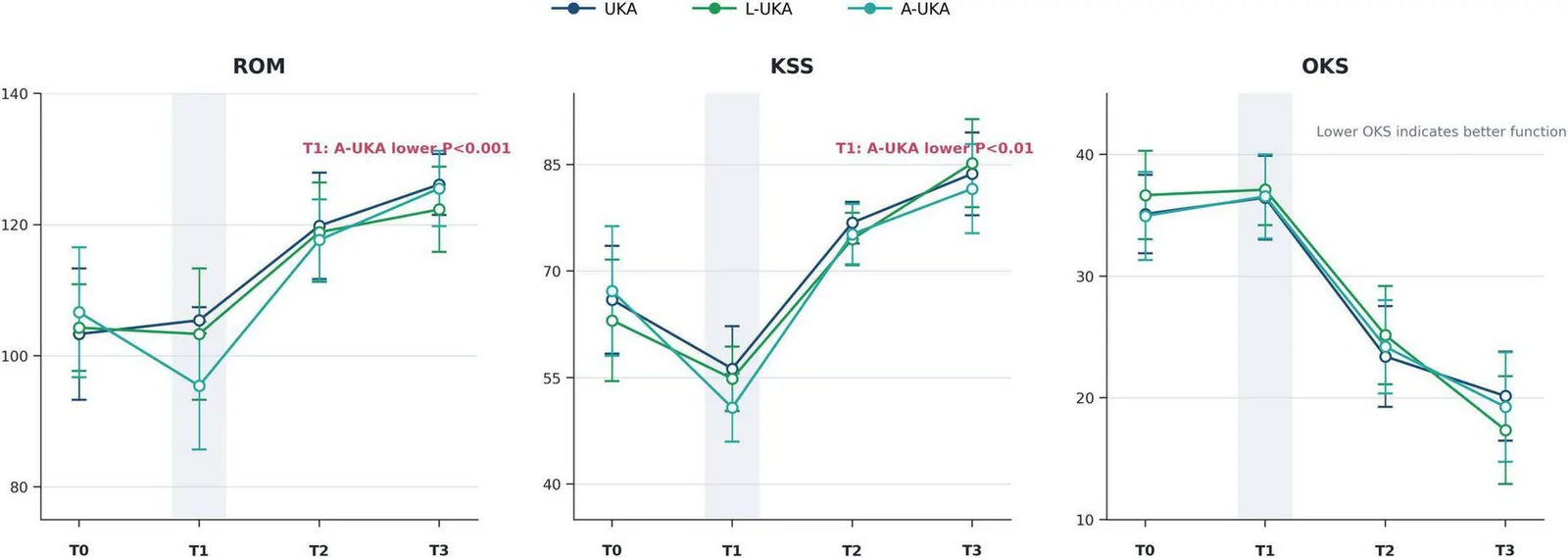
Longitudinal changes in ROM, KSS, and OKS of three groups at preoperative (T0), 1 month postoperatively (T1), 3 months postoperatively (T2), and final follow-up (T3). At T1, the A-UKA group showed significantly inferior ROM (*P* < 0.001) and KSS (*P* < 0.01) compared with the L-UKA group. Lower OKS values represent better knee function.

**FIGURE 7:**
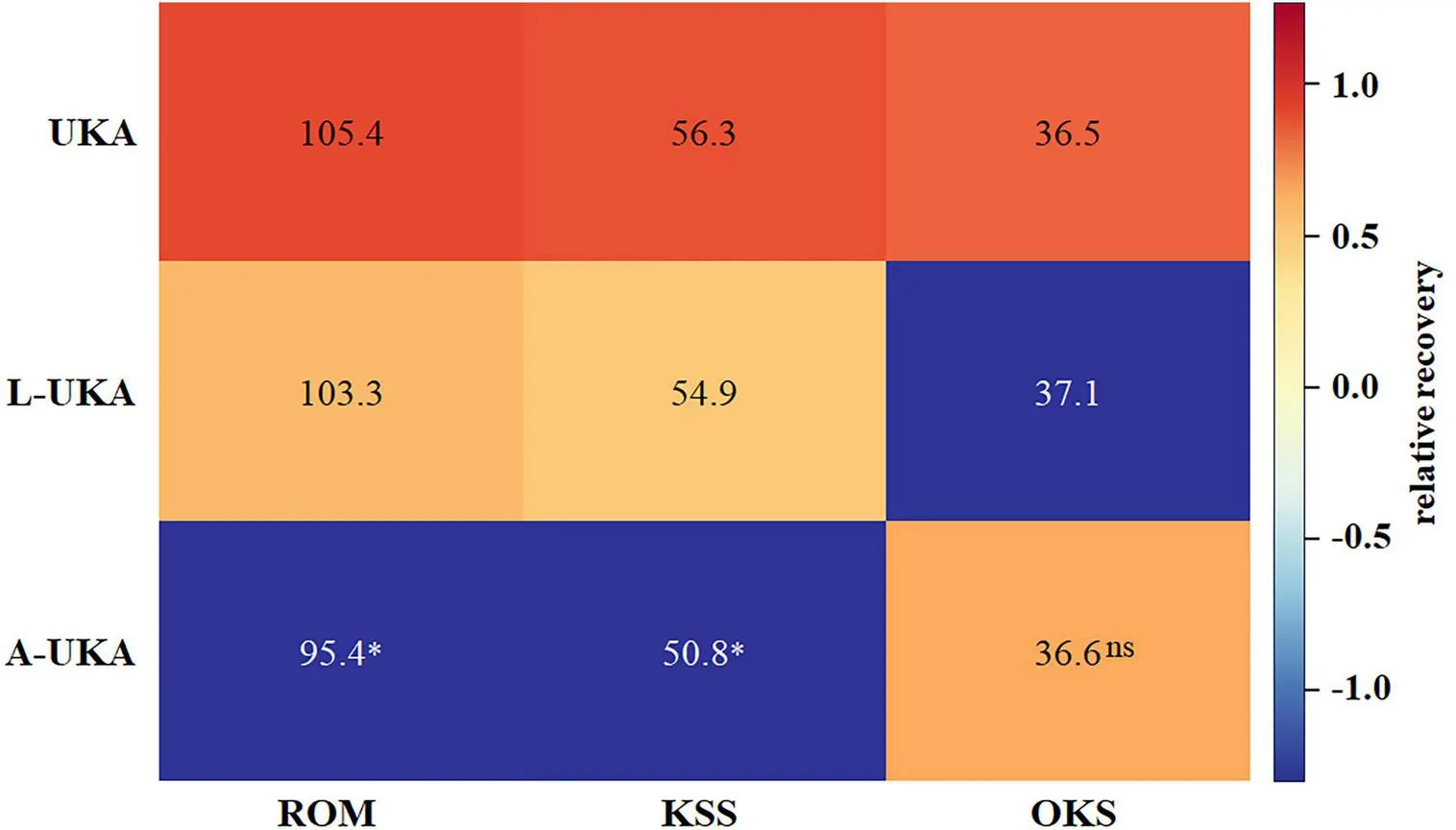
One-month postoperative ROM, KSS, and OKS across three patient groups. Superior ROM and KSS were observed in the L-UKA group compared with the A-UKA group. *P < 0.05 for L-UKA vs. A-UKA; ns, *P* > 0.05.

However, while the long-term outcomes were similar across the three groups, the postoperative functional recovery trajectories differed significantly (Figure 6). At the 1-month follow-up, the ROM (95.40 ± 9.72°) in the A-UKA group was significantly lower than the preoperative value (106.63 ± 9.91°) and lower than in the isolated UKA (105.41 ± 2.00°) and L-UKA (103.30 ± 10.03°) groups at the same time point (*P* < 0.05) (Table 4). Regarding the KSS, all groups exhibited the same trend of initially decreasing and then increasing scores. However, at 1 month postoperatively, the scores in the isolated UKA (56.25 ± 5.99) and L-UKA (54.85 ± 4.51) groups were significantly higher than those in the A-UKA group (50.75 ± 4.77) (*p* < 0.05). These trends suggest that the LARS artificial ligament has certain advantages in promoting early functional recovery in patients (Figure 7).

**TABLE 4:** Intergroup comparisons and *post-hoc* tests of clinical functional indicators (ROM, KSS, and OKS) among the three groups of patients 1 month postoperatively.

Indicator	UKA	L-UKA	A-UKA	*F*-value, *P*-value	UKA VS L-UKA *P*-value	UKA VS A-UKA *P*-value	L-UKA VS A-UKA *P*-value
ROM	105.41 ± 2.00	103.30 ± 10.03	95.40 ± 9.72	*F* = 8.39, *P* < 0.05	*P* > 0.05	*P* < 0.05	*P* < 0.05
KSS	56.25 ± 5.99	54.85 ± 4.51	50.75 ± 4.77	*F* = 6.21, *P* < 0.05	*P* > 0.05	*P* < 0.05	*P* < 0.05
OKS	36.45 ± 3.44	37.10 ± 2.92	36.55 ± 3.46	*F* = 0.23, *P* > 0.05	–	–	–

ROM: range of motion; KSS: Knee Society Score; OKS: Oxford Knee Score; *P* < 0.05 indicates a statistically significant difference.

**FIGURE 8:**
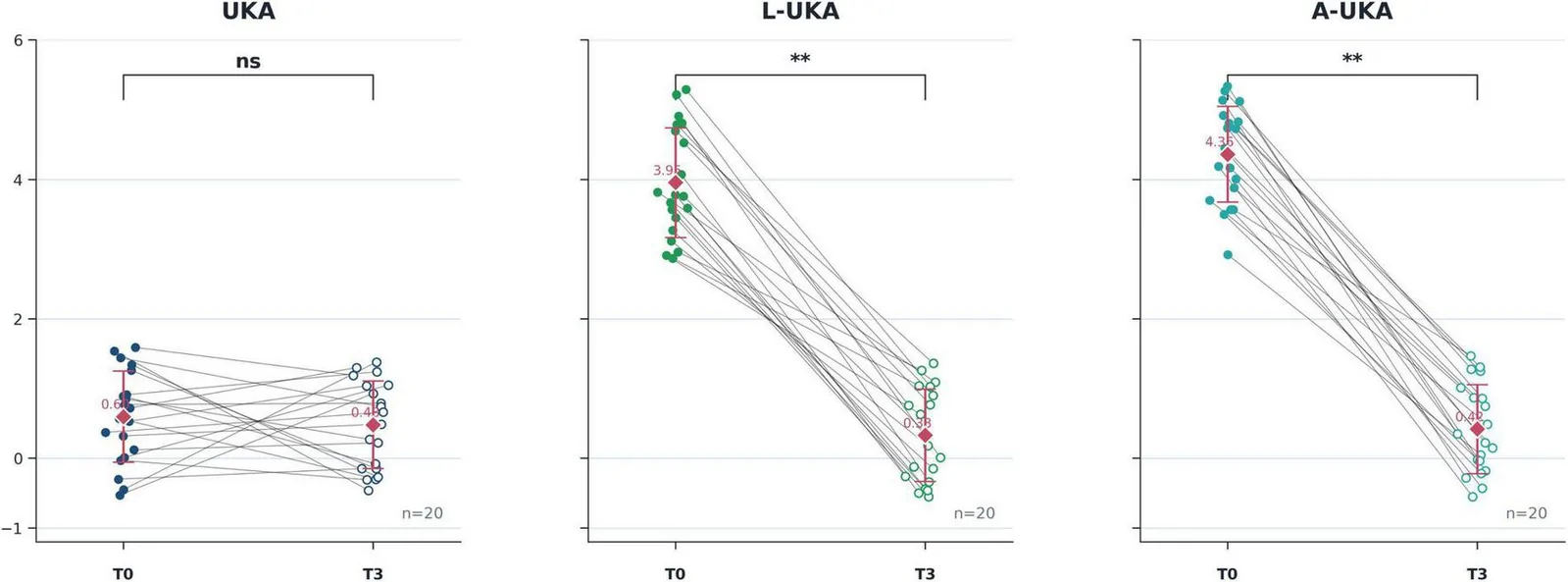
Intra-group alterations in ATT at preoperative (T0) and final follow-up (T3) across UKA, L-UKA and A-UKA groups. Individual paired patient data are linked by gray lines. No significant ATT change was detected in the UKA group, whereas obvious ATT reduction was observed in both L-UKA and A-UKA groups (*P* < 0.01). ns, *P* > 0.05; **P < 0.01.

Preoperatively, HKA and PTS values were essentially consistent across the three groups (all *P* > 0.05). At the final follow-up, these indices showed significant improvement compared to preoperative levels (intra-group comparisons, all *P* < 0.05) (Supplementary Table 3). Meanwhile, as of the final follow-up, HKA values in all three groups (UKA: 177.32 ± 1.31°, L-UKA: 178.08 ± 1.20°, A-UKA: 177.80 ± 1.20°) and PTS remained at the same level (*P* > 0.05) (Table 3). This indicates that the simultaneous addition of bone tunnel drilling and ligament reconstruction did not interfere with the accuracy of UKA prosthesis placement, and all three groups achieved satisfactory coronal alignment. Due to the loss of ACL function, the preoperative ATT in the L-UKA (3.95 ± 0.79 mm) and A-UKA (4.36 ± 0.69 mm) groups was significantly higher than that in the isolated UKA group (0.60 ± 0.65 mm) (*P* < 0.01). At the final follow-up, ATT was significantly reduced in the L-UKA (0.33 ± 0.66 mm) and A-UKA (0.42 ± 0.64 mm) groups, reaching levels comparable to those in the isolated UKA group (0.48 ± 0.63 mm) (Figure 8), suggesting that UKA combined with ACL reconstruction effectively restores sagittal stability of the knee joint.

### 3.2 Complications and prosthesis survival

At the final follow-up, no serious complications were observed in any of the three groups, including deep-seated infection, deep vein thrombosis of the lower extremities, or periprosthetic fractures. Imaging studies revealed no loosening, subsidence, or dislocation of the prosthesis, nor any radiographic progression in the lateral compartment or patellofemoral joint, defined as an increase of at least one K-L grade. In the combined surgery group, imaging and physical examinations revealed no cases of artificial or autologous ligament rupture or failure, implant loosening, symptomatic synovitis, or significant bone tunnel enlargement. In the A-UKA group, two patients reported mild pain and tightness in the tendon harvest site within the first 3 months postoperatively, which had resolved by 6 months postoperatively. Prosthesis survival rates were 100% in all three groups at the final follow-up.

## 4 Discussion

The results of this study suggest that for patients with medial compartment knee OA and ACL deficiency who have undergone strict eligibility screening, the use of a fixed-bearing medial UKA combined with ACLR using either an LARS artificial ligament or an autologous tendon yields satisfactory clinical and radiographic outcomes at mid-term follow-up. Compared with the A-UKA group, patients in the L-UKA group demonstrated superior ROM and KSS functional scores at the very early 1-month postoperative follow-up (*P* < 0.05). At the final follow-up, both combined procedures produced results that were highly consistent with those of patients who underwent isolated UKA with an intact ACL in terms of improved knee range of motion (ROM), functional scores (OKS and FJS), coronal alignment (HKA), and sagittal stability (ATT and PTS). These results suggest that TKA is not the only option for young patients with medial compartment OA who have high activity demands and definite ACL insufficiency. Fixed-bearing medial UKA combined with ACLR may offer a treatment option that better aligns with the concept of “knee preservation” by preserving bone mass, maintaining knee kinematics and promoting early functional recovery.

The core concept of UKA combined with ACLR is to address both the “source of pain” and “mechanical instability” simultaneously. UKA aims to remove degenerated cartilage and sclerotic bone in the medial compartment, restore joint space height, and improve coronal alignment of the lower limb to a moderate degree. In contrast, ACL reconstruction focuses on restoring sagittal and rotational stability to the knee joint, thereby improving the overall postoperative biomechanical environment. In theory, combining these two procedures can mitigate the risks of abnormal bearing wear and early prosthesis failure that may occur with isolated UKA in the absence of the ACL. Previously, Tian et al reported on the use of UKA combined with ACLR to treat medial compartment knee OA complicated by ACL deficiency (17–19). The results showed that, at the final follow-up (approximately 30–50 months), various functional indicators, such as ROM, OKS and Tegner scores, had significantly improved (*P* < 0.05) and were comparable to those of patients who underwent isolated UKA with intact ACLs. This confirms that this combined procedure is effective in the medium term.

However, previous studies on combined UKA and ACLR have exclusively used autologous hamstring tendon grafts. These entail prolonged recovery periods due to morbidity at the donor site and the collagen remodeling process (20, 21). This study is the first to propose using the LARS artificial ligament for combined surgery. The results showed no statistically significant differences in ROM, OKS, or FJS between the two combined surgery groups at the final follow-up. These outcomes were consistent with those of the group that underwent isolated UKA (Table 3). While functional outcomes converged at the final follow-up, early recovery trajectories diverged: at 1 month postoperatively, the L-UKA group exhibited significantly superior ROM and KSS compared to the A-UKA group (*P* < 0.05), with functional metrics gradually converging over time. This early advantage is likely due to the absence of donor site complications and the higher initial mechanical strength of the LARS graft, which is consistent with the findings of Hamido et al. that LARS confers superior early knee stability and functional recovery relative to autografts (14, 22). Consequently, we posit that UKA combined with LARS-ACLR accelerates early rehabilitation through immediate postoperative functional mobilization.

The choice of UKA prosthesis is also critical for surgical success. Mobile-bearing UKA has the advantages of reduced contact stress and superior conformity, but it requires precise soft tissue balance and knee stability (23, 24). However, ACL deficiency can increase the risk of dislocation of the polyethylene insert or abnormal motion in mobile-bearing designs (25). Although fixed-bearing UKA eliminates concerns regarding insert dislocation, it remains susceptible to aberrant tibiofemoral motion resulting from ACL deficiency. This can potentially accelerate polyethylene wear and prosthesis loosening. Kannan et al. demonstrated that fixed-bearing UKA has a significantly lower revision rate than mobile-bearing UKA (hazard ratio [HR] 1.4 [95% confidence interval (CI) 1.3–1.5], *P* < 0.001) (26). Consequently, for patients with medial compartment osteoarthritis and concomitant ACL deficiency, this study employs simultaneous ACL reconstruction with fixed-bearing medial UKA to mitigate the risk of insert dislocation and reduce surgical complexity (27). However, the long-term impact of ACL reconstruction on the polyethylene wear resistance and survival rate of fixed-bearing UKA requires validation through extended longitudinal follow-up.

Posterior tibial slope is a key anatomical parameter that influences postoperative ACL tension and ATT following UKA. Biomechanical studies have confirmed that an increase in PTS is linearly and positively correlated with ACL load (28). In patients who have undergone UKA combined with ACL reconstruction, excessive posterior tilt of the UKA osteotomy (greater than 7°) may increase the ATT and the load on the ACL graft. This increases the risk of graft laxity or even failure (29–31). In this study, we strictly controlled the PTS during UKA osteotomy, maintaining it within a safe range of 4°–5°. Statistically, at the final follow-up, no significant differences in PTS were observed among the three groups (UKA: 5.02 ± 0.91°, L-UKA: 4.53 ± 0.99°, A-UKA: 4.80 ± 1.03°; *P* > 0.05). Similarly, no significant intergroup differences were found in ATT values (*P* > 0.05), with all groups restoring to physiological levels comparable to those of the ACL-intact cohort. Due to the rigorous matching design and the deliberate intraoperative restriction of PTS within a narrow physiological safety window, the variability of PTS in our sample was minimized. Consequently, no significant linear correlation was observed between postoperative PTS and the degree of ATT reduction in the current dataset. This finding clinically validates the effectiveness of our surgical strategy: by precisely controlling PTS within 4°–5°, we avoided both excessive graft tension and increased ATT associated with an excessively large PTS, as well as abnormal joint kinematics caused by an excessively small PTS, thereby achieving consistent and stable ATT restoration across patients.

Although previous biomechanical studies have confirmed a positive correlation between PTS and ACL graft tension, our clinical practice demonstrates that precise intraoperative control of PTS within the 4°–5° range successfully eliminates the confounding effect of PTS variability on ATT outcomes, enabling all patients to achieve satisfactory restoration of sagittal plane stability. This finding supports the notion that in combined UKA and ACLR procedures, PTS should be regarded as a surgical parameter requiring “strict control” rather than treated as a “free variable.”

A primary surgical challenge in concurrent medial UKA and ACLR lies in avoiding physical conflict between the tibial bone tunnel and the UKA tibial component, particularly the prosthetic keel. Albishi et al. noted that in combined procedures, the tibial tunnel for ACLR should be positioned slightly more laterally and vertically oriented compared to conventional reconstruction to prevent graft impingement against the prosthesis and reduce stress concentration in the medial proximal tibia (32). If the tunnel is placed too close to the tibial component, not only does the risk of bone cement leakage increase, but localized stress concentration may also elevate the incidence of postoperative periprosthetic fracture (11, 33). To address this challenge, this study utilized the tibial resection surface and trial component of UKA as anatomical references to moderately adjust the guide pin angulation. Postoperative radiographic assessment confirmed the absence of tunnel–keel convergence or interference, with a preserved bone bridge of >4 mm between the tibial tunnel and the prosthetic keel. This anatomical spacing provides a sufficiently thick cancellous bone barrier to effectively reduce the risk of bone cement leakage during impaction while preserving the proximal tibial structure by preventing the superimposition of the axial compressive stress borne by the prosthetic keel and the anterior shear force transmitted through the ligament graft. There were no cases of periprosthetic fracture, cement leakage, or prosthetic loosening in the combined surgery group, which preliminarily validates the safety and precision of this tunnel positioning strategy.

It is worth noting that in combined reconstruction, we adopted a near-isometric ACL reconstruction strategy to minimize changes in graft length throughout the full range of knee flexion and extension. This strategy aims to avoid excessive tension in the graft and reduce the risk of impingement with the intercondylar fossa or UKA prosthesis structures. The native ACL does not possess a true “point of complete isometry”; therefore, clinical ACL reconstruction should aim for functional isometry. This involves minimizing changes in graft length and tension across the full range of knee motion in order to achieve good anterior stability (34). This is particularly important for UKA combined with ACLR, as abnormal graft tension can impact the kinematic environment around the prosthesis. This increases the risk of knee instability, impingement, and abnormal prosthesis loading. In this study, postoperative ROM and ATT in the combined surgery group recovered to levels similar to those in the isolated UKA group (Table 3). These results indicate that quasi-isometric tunnel placement not only helps balance graft function and prosthesis stability but also effectively improves the anterior instability of the knee caused by ACL deficiency, providing a kinematic environment closer to the physiological state for medial unicompartmental prostheses.

## 5 Limitations

This study has the following limitations: Firstly, it was a single-center retrospective study. Although a three-arm matched cohort design was employed to minimize baseline differences, the sample size allocated to each cohort remained small. The retrospective design makes it difficult to eliminate all potential unmeasured confounding factors between groups, such as patients’ specific expectations regarding physical activity or adherence, and some selection bias may still exist. Secondly, the follow-up period is currently relatively short, which makes it impossible to fully compare long-term differences in functional decline and clinical failure rates between LARS artificial ligaments and autologous tendons. Thirdly, in terms of assessing anterior knee stability, the study did not routinely use objective, quantitative tools such as the KT-1000/2000 knee measurement system. X-ray measurements of tibial anterior displacement may be subject to interference from the imaging position, resulting in measurement errors. Fourthly, regarding potential complications specific to the LARS artificial ligament, such as particulate synovitis, bone tunnel enlargement, and delayed rupture, the current follow-up duration is insufficient to draw definitive conclusions, and longer-term clinical observation is required. Fifthly, the rehabilitation plans of the A-UKA group and the L-UKA group were not exactly the same. This difference might be independent of the graft material and contribute to the functional recovery 1 month after the surgery.

## 6 Conclusion

Fixed-bearing medial unicompartmental knee arthroplasty combined with anterior cruciate ligament reconstruction using either a LARS artificial ligament or autologous hamstring tendon graft provided satisfactory mid-term clinical and radiographic outcomes in patients with medial compartment osteoarthritis and ACL deficiency. Functional and radiographic results were comparable to those of isolated UKA in ACL-intact knees. The LARS group demonstrated more favorable early postoperative functional recovery, likely related to the absence of donor-site morbidity and immediate mechanical stability. However, mid-term outcomes were similar between the two reconstruction methods. Longer-term follow-up is still required to further evaluate graft durability, potential device-related complications, and implant survivorship.

## Data Availability

The original contributions presented in this study are included in this article/Supplementary material, further inquiries can be directed to the corresponding authors.
