# Supplementary material for: Combined unicompartmental knee arthroplasty and ACL reconstruction for medial knee osteoarthritis complicated by ACL insufficiency: superior early functional recovery with LARS artificial ligament and comparable mid-term results versus autologous tendon
Source: Front Med (Lausanne). 2026 Aug 19;13:1935913. doi: 10.3389/fmed.2026.1935913 (PMC13533761; doi:10.3389/fmed.2026.1935913)
Supplement: Supplementary file 1 [file Table_1.docx]

Supplementary Material

# 1 Supplementary Tables

**Supplementary Table 1.** Consistency analysis of imaging measurements.

| Parameter | Time point | ICC | 95% CI | P value |
| --- | --- | --- | --- | --- |
| HKA | Preoperative | 0.928 | 0.825-0.957 | <0.001 |
|  | Final follow-up | 0.877 | 0.802-0.936 | <0.001 |
| PTS | Preoperative | 0.908 | 0.846-0.947 | <0.001 |
|  | Final follow-up | 0.891 | 0.789-0.943 | <0.001 |
| ATT | Preoperative | 0.841 | 0.793-0.936 | <0.001 |
|  | Final follow-up | 0.883 | 0.804-0.931 | <0.001 |

**Supplementary Table 2.** Intergroup comparisons and post-hoc tests of preoperative parameters among the three patient groups.

| **Surgical procedure** | **ROM (M±SD)** | **KSS (M±SD)** | **OKS (M±SD)** | **HKA (M±SD)** | **PTS (M±SD)** | **ATT (M±SD)** |
| --- | --- | --- | --- | --- | --- | --- |
| UKA | 103.30±10.2 | 65.95±7.60 | 35.10±3.23 | 175.06±2.67 | 9.76±2.25 | 0.60±0.65 |
| L-UKA | 104.28±6.62 | 63.05±8.58 | 36.65±3.63 | 174.24±1.90 | 10.46±2.34 | 3.95±0.79 |
| A-UKA | 106.63±9.91 | 67.20±9.11 | 34.95±3.62 | 173.13±3.02 | 11.65±2.71 | 4.36±0.69 |
| F value | 0.72 | 1.27 | 1.45 | 2.84 | 3.07 | 168.98 |
| P value | P＞0.05 | P＞0.05 | P＞0.05 | P＞0.05 | P＞0.05 | P＜0.05 |
| UKA VS L-UKA P value |  |  |  |  |  | P＜0.01 |
| UKA VS A-UKA P value |  |  |  |  |  | P＜0.01 |
| L-UKA VS A-UKA P value |  |  |  |  |  | P＞0.05 |

Note: ROM: range of motion; KSS: Knee Society Score; OKS: Oxford Knee Score; HKA: hip-knee-ankle angle; PTS: posterior tibial slope; ATT: anterior tibial translation. P < 0.05 indicates a statistically significant difference.

**Supplementary Table 3.** presents an intra-group comparison of HKA, PTS, and ATT among the three groups at the time of the final follow-up.

| **Indicator** | **procedure** | **T0(M±SD)** | **T3 (M±SD)** | **T0 VS T3 (t, P-value)** |
| --- | --- | --- | --- | --- |
| HKA | UKA | 175.06±2.67 | 177.32±1.31 | t=-3.62, P＜0.05 |
|  | L-UKA | 174.24±1.90 | 178.08±1.20 | t=-7.76, P＜0.01 |
|  | A-UKA | 173.13±3.02 | 177.80±1.20 | t=-6.46, P＜0.01 |
| PTS | UKA | 9.76±2.25 | 5.02±0.91 | t=8.73, P＜0.01 |
|  | L-UKA | 10.46±2.34 | 4.53±0.99 | t=9.72, P＜0.01 |
|  | A-UKA | 11.65±2.71 | 4.80±1.03 | t=10.80, P＜0.01 |
| ATT | UKA | 0.60±0.65 | 0.48±0.63 | t=0.53, P＞0.05 |
|  | L-UKA | 3.95±0.79 | 0.33±0.66 | t=16.84, P＜0.01 |
|  | A-UKA | 4.36±0.69 | 0.42±0.64 | t=20.19, P＜0.01 |

Note: HKA: hip-knee-ankle angle; PTS: posterior tibial slope; ATT: anterior tibial translation. T0: preoperative; T3: final follow-up. P < 0.05 indicates a statistically significant difference.
